# Moving gantry method for electron beam dose profile measurement at extended source‐to‐surface distances

**DOI:** 10.1120/jacmp.v16i2.4966

**Published:** 2015-03-08

**Authors:** Gábor Fekete, Emese Fodor, Csilla Pesznyák

**Affiliations:** ^1^ Department of Oncotherapy University of Szeged Szeged Hungary; ^2^ Department of Nuclear Techniques Institute of Nuclear Techniques, Budapest University of Technology and Economics Budapest Hungary; ^3^ National Institute of Oncology, Centre of Radiotherapy Budapest Hungary

**Keywords:** total skin electron therapy, large electron beam profile measurement, extended SSD (source‐to‐surface distance), moving gantry method

## Abstract

A novel method has been put forward for very large electron beam profile measurement. With this method, absorbed dose profiles can be measured at any depth in a solid phantom for total skin electron therapy. Electron beam dose profiles were collected with two different methods. Profile measurements were performed at 0.2 and 1.2 cm depths with a parallel plate and a thimble chamber, respectively. 108 cm×108 cm and 45 cm×45 cm projected size electron beams were scanned by vertically moving phantom and detector at 300 cm source‐to‐surface distance with 90° and 270° gantry angles. The profiles collected this way were used as reference. Afterwards, the phantom was fixed on the central axis and the gantry was rotated with certain angular steps. After applying correction for the different source‐to‐detector distances and incidence of angle, the profiles measured in the two different setups were compared. Correction formalism has been developed. The agreement between the cross profiles taken at the depth of maximum dose with the ‘classical’ scanning and with the new moving gantry method was better than 0.5 % in the measuring range from zero to 71.9 cm. Inverse square and attenuation corrections had to be applied. The profiles measured with the parallel plate chamber agree better than 1%, except for the penumbra region, where the maximum difference is 1.5%. With the moving gantry method, very large electron field profiles can be measured at any depth in a solid phantom with high accuracy and reproducibility and with much less time per step. No special instrumentation is needed. The method can be used for commissioning of very large electron beams for computer‐assisted treatment planning, for designing beam modifiers to improve dose uniformity, and for verification of computed dose profiles.

PACS numbers: 87.53.Bn, 87.53.Jw, 87.56.jf

## I. INTRODUCTION

Commissioning of horizontally directed electron beams for total skin electron therapy has been described in detail.^1^, ^2^, ^3^ As part of this procedure, uniformity checks should be performed, usually in a vertical plane at a treatment distance that is typically equal or greater than 300 cm. In clinical practice, the uniformity check means dose profile measurement, preferably at the depth of maximum dose in a tissue‐equivalent phantom.^1^, ^2^ Automated water phantoms can be used for source‐to‐surface distances up to 200 cm, but the spatial restrictions necessitate the displacement of the measuring system and the combination of partial profiles acquired this way.

The advantage of the very accurate positioning system of the water phantom cannot be utilized for longer SSD. This may introduce random uncertainties into the results. Dose profiles are often measured with diode or ion camber detectors applied on the surface of a properly sized plastic panel or phantom.^4^, ^5^, ^6^ Detailed descriptions can be found in these papers, together with profound analysis of the results. Similar grid pattern measurements were carried out in the study by Platoni et al.^(7)^ with a Markus chamber in a PMMA phantom. The patient immobilization tool was utilized for the positioning of the detector‐phantom system. Application of dosimetry film is also possible for collecting off‐axis data of a large electron field.^3^, ^8^, ^9^, ^10^ In Schiapparelli et al.,^(9)^ small pieces of radiochromic films were used in a grid arrangement to measure field uniformity on the surface of a plastic pane and for construction of isodose lines in the treatment plane. With the above‐mentioned methods, the depth of profile measurements is restricted to a specific depth, with the only exception described in the Platoni study,^(7)^ the number of measurement points is limited because of the labor‐intensive nature of such experiments. Monte Carlo calculations can be a promising alternative to profile measurements.^5^, ^11^ Verification of such a calculation needs, among others, high quality measured profile data.

In this paper, a novel method has been suggested to perform absorbed dose profile measurement for large electron beams directed at 90° and 270° angles. The profiles can be acquired at the surface or at a depth in a solid phantom with high accuracy, reproducibility, and small step width. This method is recommended for experiments where the source‐to‐surface distance is 300 cm or more.

## II. MATERIALS AND METHODS

### A. Moving detector method

**Figure 1:**
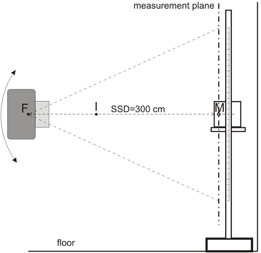
The phantom and the detector on a rack attached to a stand. F, I, and M denote the focus point, the isocenter, and the detector position, respectively.

For our measurements a Varian 2100C DX linear accelerator (Varian Medical Systems, Inc., Palo Alto, CA) was used. It has a high‐dose rate electron operating mode with 6 MeV nominal energy (HDTSe‐ mode). In this mode, the field size of the uncollimated electron beam was 36 cm×36 cm size at the isocenter. The largest possible field size has to be used. This has been achieved by rotating the gantry into the 270° direction. The largest distance applicable for treatment setup was 360 cm. A 200 cm×100 cm sized vertical frame has been built in‐house for patient positioning (Fig. 1). A horizontal rack has been attached to the vertical side poles of this frame to hold the detector‐phantom system. The detector‐phantom system consisted of a PTW M31002 thimble chamber, a M23343 Markus chamber, a UNIDOS electrometer (PTW, Freiburg, Germany), and a 25 cm×25 cm×15 cm PMMA slab phantom, that yielded full lateral scatter contribution.^(8)^ The thimble chamber was used to measure the beam profile at the depth of dose maximum, while Markus chamber has been applied to measure the profile at 0.2 cm depth in the PMMA phantom. The position of this rack — and thus the position of the detector — could be moved in this frame to any position with 0.05 cm accuracy. The distance of the phantom surface has been selected to be 300 cm from the source. The geometrical field size at this distance was 108 cm×108 cm. In this setup, the vertical profile of the large electron field could be scanned. For comparison of the results gained with the two different methods, measurement of one half of the profiles considered to be satisfactory. We used the upper half in our experiments. For full profile measurement the gantry should be rotated 180°, the stand should be repositioned at the opposite side, and the upper half profile measurement could be repeated. Measurements have been performed to establish the scatter contribution from the floor. For this purpose the lower halves of the profiles have been acquired at 90° and 110° and upper half profiles at 270° and 290° gantry angles by moving the phantom and detector up to 100 cm above and below the horizontal beam axis. Our measurements have shown agreement within 1% between the horizontal and 20° slant pair of half profiles, as well.

### B. Moving gantry method

**Figure 2:**
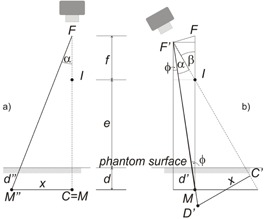
Schematic diagram of a) the moving phantom and detector, and b) the moving gantry setup. The gantry should be rotated with β degree by the moving gantry setup to have the same ray line passing through the detector that includes α degree with the central axis by the moving phantom and detector technique. β=α+ϕ holds.

**Figure 3:**
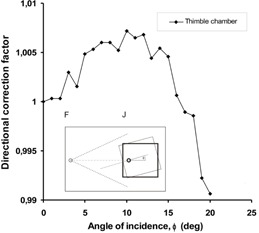
Dependence of the directional correction factor on the angle of incidence for the PTW M31002 semiflexible thimble chamber at 1.2 cm depth in a PMMA phantom positioned at 300 cm SCD (♦). The solid line represents the trend of the measured values.

Profile measurements were performed in another way, as well. By this moving gantry method, the effective point of measurement was fixed on the central axis in the vertical plane of measurement that is at 300+d cm distance from the F focus point (Fig. 1), where d is the desired depth of measurement. Let us assume, that the point of interest is at x cm distance from the central axis, according to Figs. 2(a) and (b). Rotating the gantry with β degree, the ray line including α angle with the central axis will pass through the detector (M in Fig. 2(b)). After some simple trigonometric considerations, α can be expressed as the function of β:

(1) $$\alpha =\beta -arctg\left(\frac{f\cdot \text{sin}\beta }{f\cdot \text{cos}\beta +e+d}\right)$$

where *ƒ* is the source‐to‐isocenter distance, *e* is the isocenter‐to‐phantom surface distance, and *d* is the depth of the effective point of measurement for the specific ion chamber in use. The distance of the chamber from the source and the thickness of phantom material crossed by the electrons moving along the ray line decrease relative to the ‘classical’ moving detector setup, shown in Fig. 2(a). The phantom scatter component also changes due to the different angle of incidence onto the phantom surface. For this difference, correction has to be applied:

(2) $$R^{\prime }(\phi )=R(\phi )\cdot C_{isq}\cdot C_{att}\cdot C_{obl}$$

where $R^{\prime }$ and *R* are the corrected and uncorrected readouts, $C_{\text{isq}}$ is the inverse square correction factor, $C_{\text{att}}$ and $C_{\text{obl}}$ take into account the difference in absorber thickness and angle of incidence, respectively. The $C_{\text{att}}\cdot C_{\text{obl}}$ factor has been determined in this study experimentally for the specific depths of interest and source‐to‐surface distance as follows. The ion chamber was inserted into the phantom with its effective point of measurement at the depth of interest, and was positioned on the central axis of the beam with the phantom surface perpendicular to this axis. The phantom was turned around an axis going through the effective point of measurement and perpendicular to the central axis of the beam, while keeping the chamber on the beam axis (cf insert in Fig. 3). This way the inverse square correction was omitted. Readouts were collected at different phantom angles (ϕ) and were normalized to the value taken at perpendicular incidence. It is worth to note that in moving gantry measurement setup, ϕ=β−α holds. In this setup, the chamber has been positioned at the depth in consideration, the gantry angle was changed between 0° and 20° for the thimble chamber, and between 0° and 30° for the Markus chamber.

The inverse square correction factor, $C_{\text{isq}}$, between the two measuring setups can be expressed with the lengths of the line segments drawn from the source to the point of interest:

(3) $$C_{isq}={\left(\frac{F^{\prime }M}{FM^{\prime \prime }}\right)}^{2}=\frac{{\left(f\text{cos}\beta +e+d\right)}^{2}+f^{2}{\text{sin}}^{2}\beta }{{\left(f+e+d\right)}^{2}}{\text{cos}}^{2}\alpha$$

By application of the above‐derived formalism, the measurement carried out at a gantry angle β can be transformed into a measurement performed in the normal setup at x cm distance from the central axis, where

(4) $$x=(f+e+d)\cdot tg\left(\beta -arctg\left(\frac{f\cdot \text{sin}\beta }{f\cdot \text{cos}\beta +e+d}\right)\right)$$

The gantry angle can be changed in 0.1° increment, which corresponds to 0.35−0.37 cm step width at 300 cm, depending on the distance from the central axis. The reproducibility of the gantry angle has been checked in this study and found to be better than 0.1 cm at 300 cm distance. Profiles were measured by changing β from 270° to 290° with 1° increment. In the ‘classical’ setup, the X coordinates of the measuring points were calculated from Eq. (4), thus ensuring that the measurements had been carried out along the same ray line, making comparison straightforward.

During the profile measurements, care should be taken of the currents created in the chamber cable, connector, and extension cable.^12^, ^13^, ^14^, ^15^ For electron fields, the cable effect is directly proportional with the irradiated cable length and leakage current predominates.^(13)^ At each measurement point the readouts taken with positive and negative chamber polarity have been averaged. The chamber cable was fixed to the phantom holder in horizontal position, and the extension cable was placed outside the direct beam. The upper half of the electron beam was measured with both techniques, so the amount of irradiated chamber and extension cable was the same, and the cable effect assumed to be identical. No correction has been applied to compensate for this effect.

## III. RESULTS & DISCUSSION

It has been found experimentally in this study, that the inverse square law was valid between 290 and 310 cm, with accuracy better than 0.5%. Thus, Eq. (3) could be applied to calculate $C_{\text{isq}}$. The measured values of $\frac{R(\phi )}{R(\phi =0)}=C_{att}\cdot C_{obl}$ as the function of the angle of incidence, ϕ, and SSD for the thimble chamber are shown in Fig. 3. Because of the cylindrically symmetric nature of the thimble chamber, $C_{\text{obl thimble}}=1.0$ has been assumed. The values measured at 300 cm have been fitted with the following formula:

(5) $$\frac{R(\phi )}{R(\phi =0)}=1-0.00018\cdot \phi^{2.0}$$

From Eq. (5), the necessary correction factor can be derived by applying it to α and ϕ:

(6) $$C_{att,thimble}=\frac{1-0.00018\cdot \alpha^{2.0}}{1-0.00018\cdot \phi^{2.0}}$$

**Figure 4:**
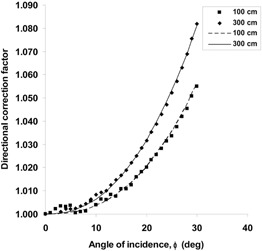
Dependence of the directional correction factor on the angle of incidence for the PTW M23343 Markus chamber at 0.2 cm depth in a PMMA phantom positioned at 100 cm (▪) and 300 cm SCD (♦). Broken and solid lines represent best fit at 100 cm and 300 cm SCD, respectively.

For the Markus chamber, the measured dependence on the angle of incidence can be seen in Fig. 4. This dependence can be fitted by the following equation:

(7) $$\frac{R(\phi )}{R(\phi =0)}=1+a\cdot \phi^{b}$$

where *R*(ϕ) is the readout for the angle of incidence, ϕ. In Eq. (7), α=0.0000114, b=2.5 for 100 cm SSD, and α=0.0000325, b=2.3 for 300 cm SSD. The corrections due to attenuation and oblique incidence cannot be separated in this case. According to equation (5b), the directional correction factor for the Markus chamber is

(8) $$\begin{array}{l} C_{att,obl,Markus}=\frac{1}{1+a\cdot \phi^{b}} \end{array}$$

This factor has to be applied in both ‘classical’ and moving gantry setup in order to compare readouts. When measuring in the moving gantry setup, the gantry angle increases up to 290° (20° in Fig. 2), while the angle of incidence, ϕ, increases to only 6.6° relative to the surface normal. For this value of ϕ, Eq. (8) yields a value of 1.0026 at 300 cm SSD. From this result, it is clear that the attenuation and obliquity correction would have been negligible for the Markus chamber in the moving gantry setup, but had to be applied in the moving detector setup.

**Figure 5:**
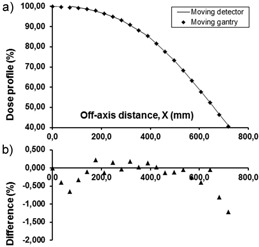
Off‐axis half profile of a $108\times 108\;{\text{cm}}^{2}$ horizontal electron beam measured at 300 cm SSD: a) by moving the semiflexible detector‐phantom system across the beam (—), and (b) by moving the gantry (♦). The center of the ion chamber is at 1.2 cm depth. Percentage differences are shown with solid triangles.

**Figure 6:**
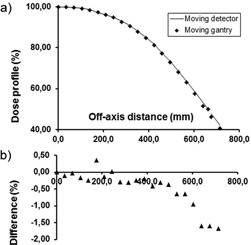
Off‐axis half profile of a $108\times 108\;{\text{cm}}^{2}$ horizontal electron beam measured at 300 cm SSD: a) by moving the Markus chamber‐phantom system across the beam (—), and (b) by moving the gantry (♦). The effective measuring point of the ion chamber is at 0.2 cm depth. Percentage differences are shown with solid triangles.

**Figure 7:**
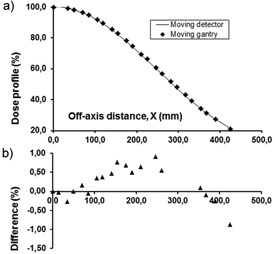
Off‐axis half profile of a $45\times 45\;{\text{cm}}^{2}$ horizontal electron beam measured at 300 cm SSD: a) by moving the semiflexible detector‐phantom system across the beam (—), and (b) by moving the gantry (♦). The center of the ion chamber is at 1.2 cm depth. Percentage differences are shown with solid triangles.

The profiles measured with the thimble chamber at the depth of dose maximum are shown in Fig. 5. After the application of corrections detailed above, the difference between the two profiles was smaller than 0.5% over the whole measuring range. The profiles measured at 0.2 cm depth with the Markus chamber are shown in Fig. 6. In this case the maximum difference was 1.5% in the outermost measuring point. The penumbra measured in the moving gantry setup is steeper than that with the ‘classical’ one. The difference in the X coordinates of the 50% profile points is 0.95 cm, which distance corresponds to 0.32 cm projected back to 100 cm distance from the source. The Markus chamber is not the best choice for profile measurement, but considering the shallow measuring depth and the dimension of its sensitive volume relative to the size of the beam penumbra, its use may be a reasonable compromise when measurement of the beam profile on the phantom surface is required. In the 60% to 100% region, the agreement between the profiles taken with the Markus chamber in the different setups is better than 0.8%. To further investigate the performance of the method in the full penumbra region, the half profile of a 45 cm×45 cm electron field at the depth of maximum dose has also been measured (Fig. 7) with the thimble chamber. The maximum difference between the two profiles was 0.7%.

## IV. CONCLUSIONS

A novel method has been proposed for electron beam in‐plane profile measurement at a source‐to‐surface distance equal to or larger than 300 cm. Obliquity and attenuation correction factors had to be determined at the phantom depth and SSD in consideration to reproduce ‘classical’ profiles. The depth of measurement can be selected at any depth typical in total skin electron therapy, but directional corrections should be determined experimentally for those depths. The size of the slab phantom can be small, because full lateral scatter can be achieved easily.^(8)^ The instrumentation needed for this method is readily available at any radiotherapy department. The positional reproducibility of the measuring points is high, thus the method is applicable for quality control purposes, as well. Due to this positional reproducibility and the arbitrarily selectable measuring depth, the verification of beam calculation models for treatment planning^5^, ^11^ at large source‐to‐surface distance, or testing secondary flattening filters designed to improve beam uniformity is also possible with this method.

With the moving gantry method, large horizontally directed electron beam profiles can be measured at arbitrary depth in a plastic phantom at arbitrary source‐to‐surface distance with small step width and with high accuracy. The floor scatter is not included in the profiles collected this way because the point of measurement is far from the floor. The authors intend to generalize the above formalism in a separate study to make it suitable for profile measurements of declined large electron beams, those are important in the course of the commissioning of total skin electron therapy techniques.
